# Correlation between oral health and quality of life among the elderly in Southwest China from 2013 to 2015

**DOI:** 10.1097/MD.0000000000010777

**Published:** 2018-05-25

**Authors:** Xun Sheng, Xia Xiao, Xiaoxiao Song, Lei Qiao, Xihong Zhang, Hua Zhong

**Affiliations:** a Department of Stomatology, The Affiliated Stomatological Hospital of Kunming Medical University; b Department of Community Health, Kunming Medical University School of Public Health; c Department of Ophthalmology, First Affiliated Hospital of Kunming Medical University, Kunming, China.

**Keywords:** dental caries, dentition, gingivitis, life quality, periodontitis

## Abstract

The aim of this study was to evaluate the oral health among the elderly in Southwest China and to analyze the correlation between common oral diseases and quality of life (QOL) in the same population, thus providing recommendations to improve their oral health and life quality.

Elderly people (>60 years’ old) were randomly recruited into our study, and we performed oral health examinations and diagnoses, using the Community Periodontal Index (CPI) to assess the periodontal condition, and Oral Health Impact Profile-14 (OHIP-14) to score life quality associated with oral health. Then we analyzed correlations between oral health and QOL as well as body mass index (BMI).

A total of 687 subjects participated in our study and 212 (30.9%) were diagnosed with gingivitis or subgingival calculus, 291 (42.4%) with moderate chronic periodontitis, 136 (19.8%) with severe chronic periodontitis, 514 (74.8%) with dental caries, and 648 (94.3%) with dentition defects. A total of 653 (95.1%) qualified OHIP-14 scores were collected, with a median score of 13. The scores of the severe and moderate periodontitis were similar to the dentition defects, but higher than the scores for gingivitis and subgingival calculus. Considering the most common side effect, 11% of the subjects with severe chronic periodontitis were reported to be “unsatisfied with eating,” and 48.4% of the participants with dentition defects complained about “troubles with pronunciation.” A logistic regression analysis revealed that underweight (BMI <20) correlated with dental caries (odds ratio [OR]: 0.167, *P* = .040) and dentition defects (OR: 0.119, *P* = .016).

The general oral health condition was poor among the elderly in Southwest China. Periodontitis and dentition defects have considerable negative effects on the QOL among this population.

## 1 Introduction

Oral diseases in the Chinese elderly are mainly composed of periodontal disease, dental caries, dentition defects, and other conditions.^[1]^ Periodontal disease is a term generally used to describe specific diseases that affect the supporting tissues and is divided into gum diseases and periodontitis, which affect the deep layer of the periodontium (periodontal ligament, alveolar bone, and dental cementum). The periodontal disease induces periodontal tissue destruction and alveolar bone absorption, causing the teeth to drop out and in fact it has become the main reason for the loss of teeth in elderly Chinese people.^[2]^ Dental caries is a progressive lesion of the tooth enamel influenced by multiple oral factors, manifested as demineralization of the inorganic part and degradation of the organic part.^[3]^ As the lesion progresses, dental caries can gradually induce the formation of holes, residual crowns, residual roots, and eventually teeth loss.^[4]^ On account of the oral environmental change in the elderly (such as less secretion of saliva), along with the degeneration of cognitive sensitivity, unnoticed dental caries can proceed rapidly and as notable symptoms show up, they are unlikely to preserve the involved teeth. Hence, the 2 diseases can cause dentition defects and even tooth loss, bringing trouble to the elderly regarding mastication and pronunciation, thus affecting their life quality.

Quality of life (QOL) refers to the feelings of individuals in a certain cultural environment and is associated with personal goals, expectations, standards and concerns, and includes physiological health, the mental state, independency, social relationship, personal beliefs, and the environment.^[5]^ QOL not only provides clinical doctors and researchers with more information about the disease, but also quantifies the influence of a certain disease on physical, psychological, and social aspects for patients, which is beneficial for health risk factor tracing, treatment selection, and prognosis monitoring, and this supports the concept of modern health and the transition of medical models.^[6]^ In oral medicine, because oral health-related QOL (OHRQOL) can also function as an assessment of oral diseases on physical, psychological, and social aspects for patients, it has recently been paid much more attention and taken into consideration in practice.^[7]^

OHIP-14 is a widely used scale in the OHRQOL area, whose reliability and validity have been intensively verified. The OHIP-14 scale has 14 items, divided into 7 domains: functional limitation, physical pain, psychological discomfort, physical disability, psychological disability, social disability, and handicap. Several studies have successfully applied this scale to evaluate the effects of chronic periodontitis on life quality in the elderly.^[8,9]^

At present, large sample size studies on oral health are scarce in China and our study investigated the epidemiology of oral diseases among the elderly in Southwest China by evaluating their current oral health status as well as any correlations between common oral diseases and QOL to produce a regional survey on present oral health awareness in a Chinese province.

## 2 Patients and methods

### 2.1 Investigation population

The study was approved by our institutional review board and informed written consent was obtained from all of the recruited patients. Population data were collected from 13 districts in Southwest China (Panlong, Wuhua, Xishan, Guandu, Chenggong, Jinning, Fumin, Songming, Anning, Dongchuan, Xundian, Shilin, Luquan) from September 2013 to January 2015.

Inclusion criteria were: ≥60 years’ old with self-care ability and could move freely; local resident; conscious; able to communicate and willing to participate in the study. Exclusion criteria were: local resident <2 years in Kunming City; receiving seizure, thyroid, or chemotherapy/radiation medications; positive HIV status; and denial of participating in the study. We enrolled patients based on the records of their household register in the hospitals and the inclusion and exclusion criteria defined for our study.

### 2.2 Oral Examination

**Figure 1:**
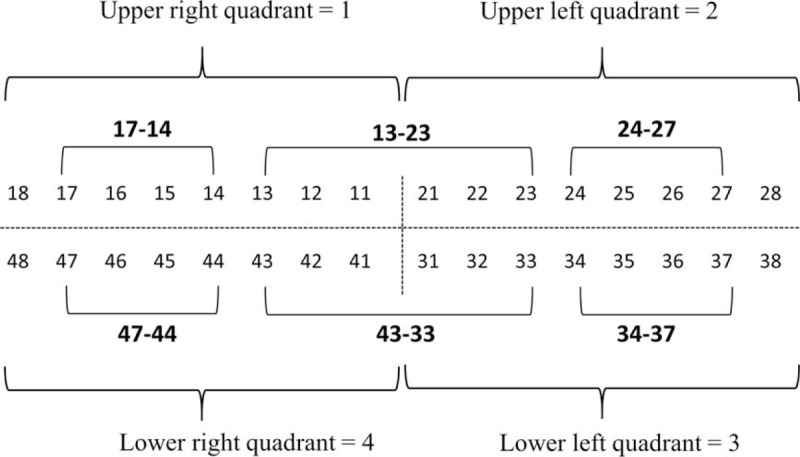
World Dental Federation classification of teeth in 4 quadrants with 8 teeth per quadrant numerated from the incisors to the molar teeth. The bold numbers indicate the teeth zones, which have been analyzed in the study.

**Figure 2:**
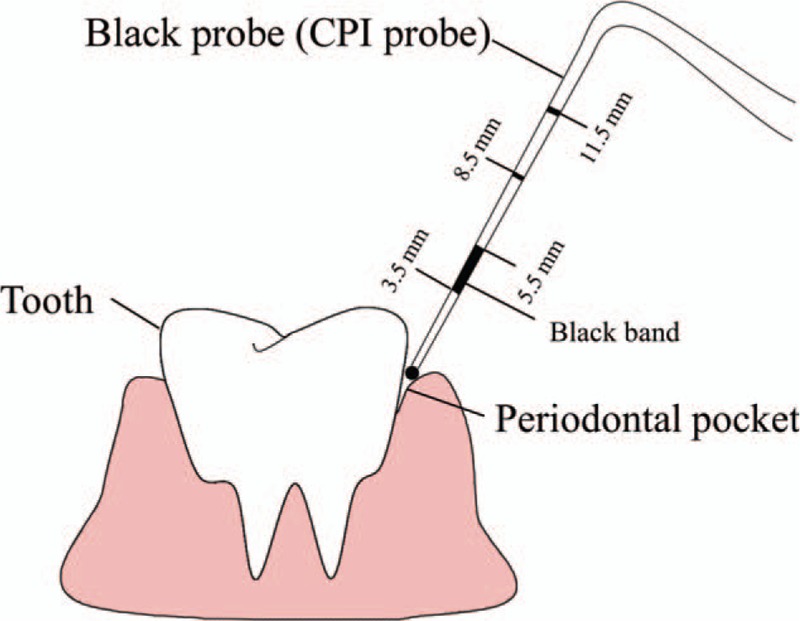
Dental CPI probe used for measuring periodontal pocket depths. CPI = Community Periodontal Index.

Two dental doctors who had unified test standards after training, to reduce the error caused by standard deviation, were responsible for the oral examinations of all participants. Detailed oral examination and diagnostic standards were as follows: the periodontal disease was diagnosed using the Community Periodontal Index of treatment needs (CPITN).^[10]^ The teeth have been categorized into 4 quadrants (upper right and left and lower right and left, with 8 teeth in each quadrant according to the World Dental Federation (FDI) guideline.^[11]^ Periodontal conditions, including gingival bleeding, subgingival calculus, and depth of periodontal pocket, were evaluated in 6 zones: 17-14, 13-23, 24-27, 47-44, 43-33, and 34-37 (Fig. 1). Each patient was diagnosed according to CPI criteria with a CPI probe (Fig. 2) from Code 0 to Code 4 (Code 0: healthy periodontal conditions; Code 1: gingival bleeding on probing; Code 2: calculus and bleeding; Code 3: periodontal pocket 4–5 mm; and Code 4: periodontal pocket ≥6 mm).^[10]^

Dental caries status was assessed using criteria of the Decayed-Missing-Filled Teeth index (DMFT index) recommended by the World Health Organization.^[12]^ Oral examination should be focused on visual examination, assisted by probing, mainly evaluating the color change of the surface and morphology defects such as a chalk color or a brown patch on the surface, black tooth fossa, ink change on the groove and edge of the occlusal surface, dark brown cavity, and residual root or crown.

### 2.3 Dentition status and treatment demand

We checked and recorded coronary dental caries, root dental caries, secondary dental caries, history of filling a tooth, dentition loss and reason for dentition loss, abutment stat,e and the treatment program for each tooth. Dentition defects refer to different numbers of natural teeth missing in the maxillary and mandible, whereas dentition loss means that no natural teeth or tooth roots are left on the entire dental arch.

### 2.4 Questionnaire survey

The general demographic data and OHIP-14 scale were collected by questionnaire survey.^[13]^ General demographic data included sex, age, nationality, residence, living conditions, education level, occupation, monthly income, and marital status, and so on. The OHIP-14 scale contained 7 dimensions and 14 items, namely 14 questions. Each question had 5 answers about frequency, which were respectively “very often,” “often,” “sometimes,” “seldom,” and “never,” The participants could choose one answer under investigation and one explanation. The score of OHIP-14 ranged from 0 to 56. A higher score suggested a worse life quality associated with poor oral health.

### 2.5 Statistical analysis

We used Epidata software to establish a data input template, and adopted a double input method for all valid questionnaires. We insured the integrity of data by a data verification program. The score of OHIP-14 scale are presented as median and quartile intervals. The methods of statistical analysis included logistic regression, descriptive statistics, Pearson *χ*^2^ test, Fisher *χ*^2^ test, and rank sum test. Inter-rater reliability has been determined by Cohen Kappa coefficient calculation. *P* < .05 was considered to be statistically significant.

## 3 Results

### 3.1 General demographic data

This study investigated 700 participants, 687 of whom completed valid questionnaires (98%). Among the respondents, the number of males was slightly higher than females; 60- to 70-year-old subjects accounted for the majority (68.7%); most of them were from the Han nationality (93.7%); the education level was high school or secondary school among most participants (37.3%), followed by junior college and above (26.8%); monthly income was relatively high (>1500 yuan, 75.2%); most of them were married (91.8%) and had 2 to 5 family members (93.8%) (Table 1).

**Table 1:**
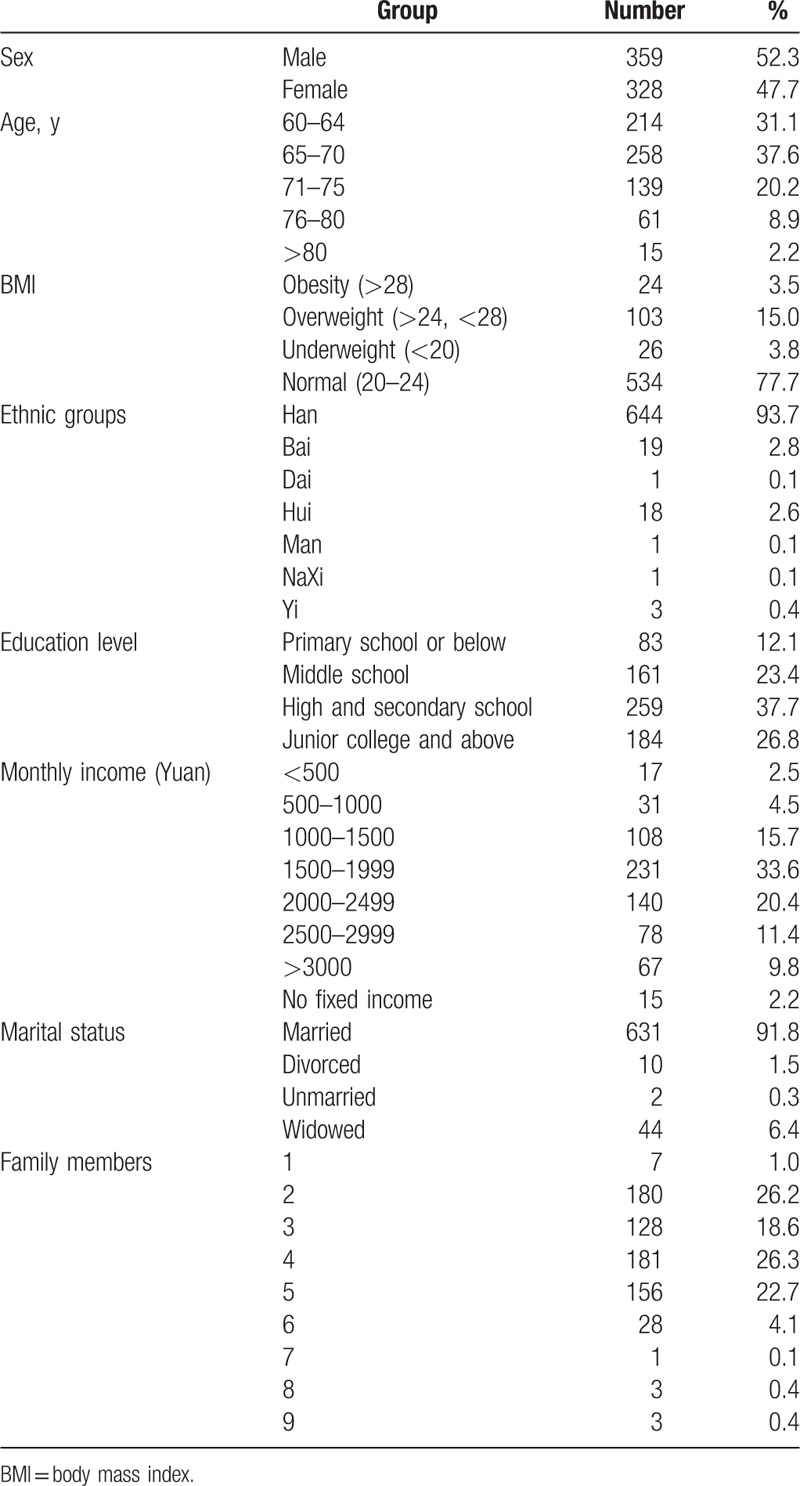
General demographic data.

### 3.2 Oral diseases

Kappa values of interexaminer agreement were 0.75 to 0.80. The results of the oral health evaluation for the 687 respondents were reported as a serious problem, and the numbers of subjects with gingivitis or subgingival calculus, moderate periodontitis, and severe periodontitis were reported to be 212 (30.9%), 291 (42.4%), and 136 (19.8%), respectively. Only 2 subjects had completely healthy teeth and the remaining 41 had no experiences or no access to CPI examination. As shown in Table 2, both male and female participants were mainly in the moderate stage of periodontitis. When compared with females, periodontitis was more likely to develop into the severe stage in male subjects (30.9% vs. 35.5%). Moreover, we found that with an increase in age, the severity of the disease also increased gradually: subjects aged 60 to 64 years were mainly diagnosed with gingivitis or subgingival calculus (45.5%), those aged 65 to 70 years and 71 to 75 years were diagnosed with moderate periodontitis (48.4% and 58.3%), whereas the oldest subjects (76–80 years) were likely to be diagnosed with either severe periodontitis or moderate periodontitis (respectively 32.6% and 37.0%). There were only 10 participants above 81 years of age, which limited the interpretation for patients in this group. Considering the education level, participants of the middle school group were more likely to be diagnosed with gingivitis or subgingival calculus, whereas the others mostly had moderate periodontitis. We further analyzed the correlation between monthly income and the prevalence of each condition, and found that as income increased, the prevalence of moderate or severe periodontitis decreased: the prevalence rate of the low-income (<500 yuan) subjects was 90.9%, whereas the rate of the high income (>1500 yuan) subjects was 62.7%. Two healthy participants were found in the high-income group (>1500 yuan). Finally, according to Pearson *χ*^2^ test, there were significant differences (*P* = .043) between male and female subjects, among subjects of different ages (*P* *<* .001), education levels (*P* *<* 0.001), and monthly income (*P* *=* .001).

**Table 2:**
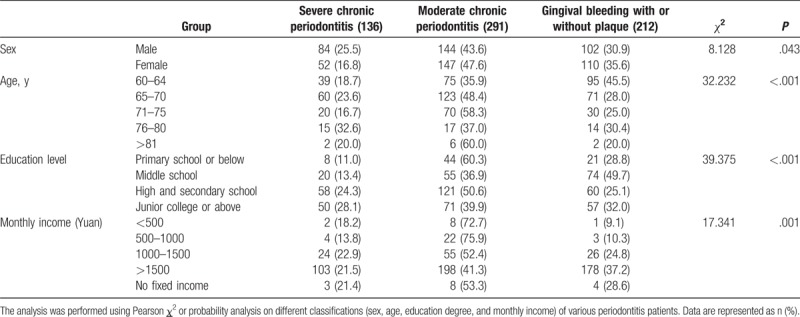
Comparison of correlations between different stages of periodontitis and the baseline information of enrolled patients.

### 3.3 Dental caries, tooth loss and filled teeth

There was a significant difference between the age groups regarding dental caries and dentition defects (*P* < .001). Among 687 respondents, 514 (74.8%) had dental caries. The prevalence of dental caries in the older subjects (>75 years’ old) was lower than those aged 60 to 75 years. In addition, 648 (94.3%) of them had dentition defects, the average missing teeth were 11.21, and 31 (4.5%) had dentition loss. The rate of dentition defects in males and females was comparable (93.9% and 94.8%). Similar to dental caries, the dentition defect rate was lower in the older subjects (>75 years’ old) compared with those aged 60 to 75 years (Table 3).

**Table 3:**
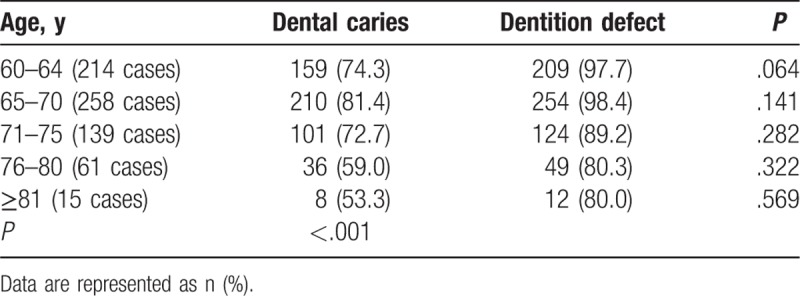
The prevalence of dental caries and dentition defects in subjects of different age groups.

### 3.4 OHIP-14 scores

**Figure 3:**
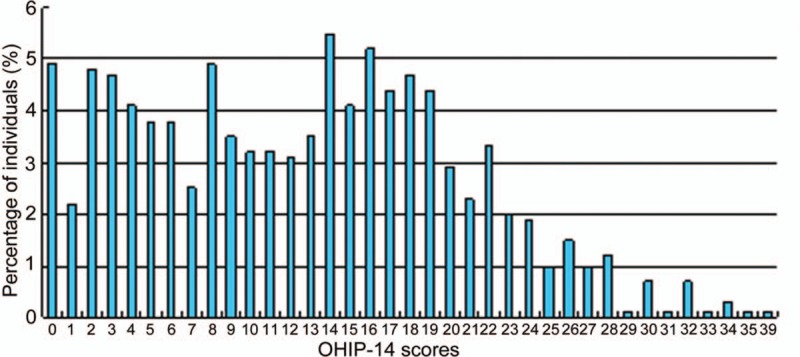
Distribution of the OHIP-14 scores. OHIP-14 = Oral Health Impact Profile-14.

In total we collected 653 (95.1%) qualified OHIP-14 scores from 687 respondents. As shown in Figure 3, the median score was 13 and the highest score was 39. We further analyzed the correlation between oral diseases and QOL. Participants with severe and moderate chronic periodontitis had similar OHIP-14 scores, with a median score of 15.5 (10, 19) and 14 (7, 18), respectively. In comparison, the OHIP-14 scale scores of gingivitis and subgingival calculus participants were relatively lower, with a median score of 6 (2.75, 11.5) and 8 (2, 14), respectively. It was noted that participants with severe periodontitis complained mostly about “being unsatisfied with eating” (11.0%), whereas patients with moderate periodontitis commonly reported (6.5%) “trouble pronouncing words,” whereas patients with gingivitis and subgingival calculus (5.7%) were “uncomfortable eating foods” (Table 4). When comparing the negative effects of moderate and severe chronic periodontitis, we concluded that severe periodontitis had more negative effects than moderate periodontitis on the following items: “painful aching in the mouth,” “unsatisfactory diet,” “being irritable with others,” and “felt life is less satisfying,” with statistical significance being attained (Table 4).

**Table 4:**
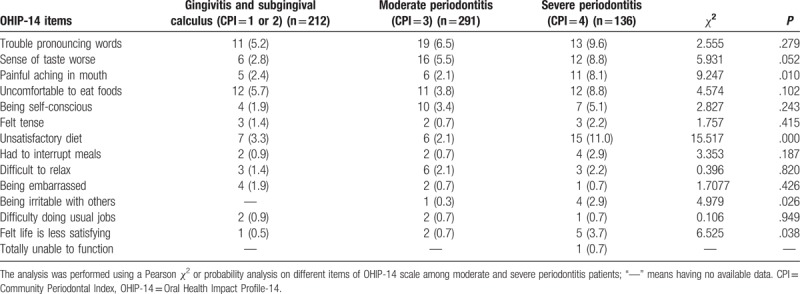
The effect of periodontitis stages in subjects on OHIP-14 Chinese version evaluation items.

Considering the effect of dentition defect/loss of life quality, we found that the median OHIP-14 score in the elderly with complete dentition was 3 (0.25, 5.75), the median score of patients with dentition defects was 13 (6, 18), and the median score of patients with dentition loss was as high as 23 (16, 28). Concerning the item of “trouble pronouncing words,” both dentition defects and loss patients reported the most pronounced negative effects (7.1% and 48.4%) (Table 5). In addition, dentition loss patients experienced more negative effects than those with dentition defects in the following items: “trouble pronouncing words,” “sense of taste worse,” “unsatisfactory diet,” “had to interrupt meals,” and “felt life is less satisfying,” with statistical significance being achieved (Table 5).

**Table 5:**
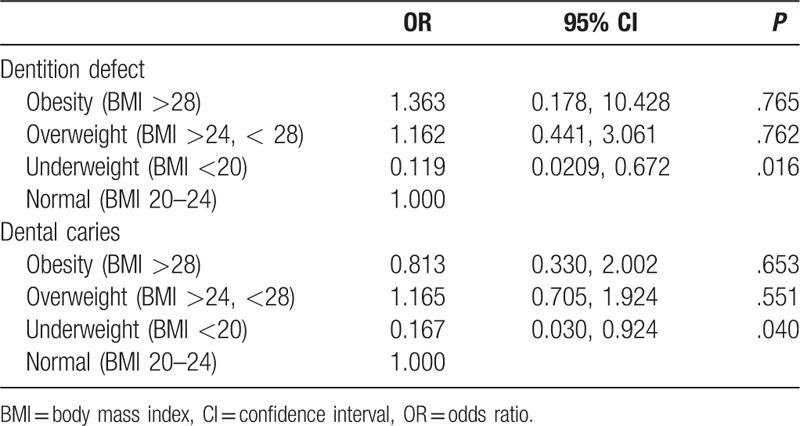
The effect of dentition defects or dentition loss on OHIP-14 item scores.

### 3.5 Body mass index (BMI) was associated with dentition defects and dental caries

Finally, we analyzed correlations between oral health and body weights of the subjects in a logistic regression analysis. The results showed that dental caries and dentition defects were significantly reflected in subjects with BMIs <20 (odds ratio [OR]: 0.167, *P* = .040 and OR: 0.119, *P* = .016, respectively) (Table 6).

**Table 6:**
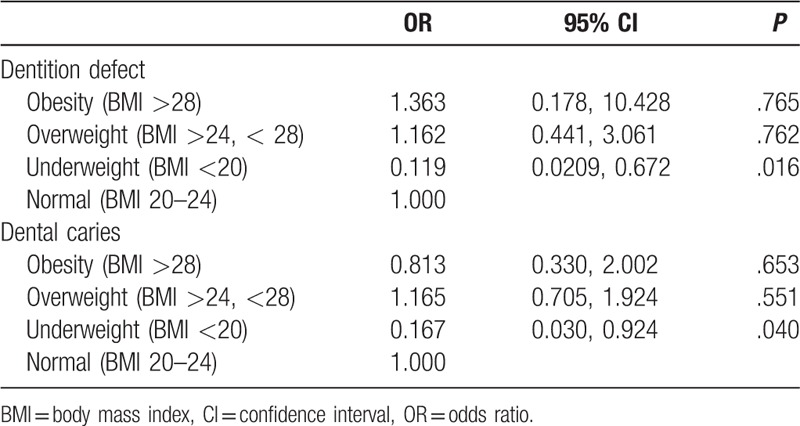
A logistic regression analysis of associations between BMI and dentition defect and dental caries.

## 4 Discussion

Oral diseases, especially periodontal disease and dental caries, are still major health problems in the world today.^[14]^ Our study showed that the oral health of the elderly in Southwest China was worrying. Among 687 respondents, only 2 subjects had completely healthy teeth (0.3%), whereas the prevalence of gingival bleeding, dental caries, and moderate or severe periodontitis were 30.9%, 42.4%, and 19.8%, respectively. With an increase in age, the main disease type changed from gingivitis and dental caries to severe periodontitis, which led to severe oral disease consistent with our expectations. Because the dental plaques of the elderly (>65 years’ old) could not be well controlled, dental caries and dentition defects were more common in these people, coupled with a worse systemic health condition, and decreased disease resistance or self-repair capacity. All of these factors resulted in a vicious cycle, causing severe periodontitis. Similarly, as income increased, we found a gradual decrease in the prevalence of moderate and severe periodontitis, suggesting a close relationship between individual income and oral health. Because of insufficient income, the elderly tended to put general health as a first priority, paying more attention to the health of respiratory, circulatory, digestive, and neural systems, while ignoring the importance of oral health. For example, we found that most elderly people thought loose teeth was normal and not worthy of attention! Therefore, this kind of unadvisable concept of oral health could also be related to the high prevalence of periodontitis among these subjects.

Our study also found that the prevalence of dental caries and dentition defects was high in the elderly in Southwest China, whereas the corresponding repair was insufficient. In all respondents, up to 74.8% suffered from dental caries, up to 94.3% had dentition defects, and 45.4% and 49.5% of these participants had no prosthesis in the maxillary and mandible. However, the rates of partial denture wearing in the maxillary and mandible were 34.9% and 33.8%, respectively. The low repair rate, in addition to the relatively poor income of old subjects, was also related to the traditional health concept and lack of attention to oral health care. Besides, considering the average life expectancy was <70 years, >75 years of age should belong to the high-age group. Our results demonstrated that the prevalence of dental caries and dentition defects were significantly lower in the high-age group than in the 60- to 75-year-old group, which reflected the positive correlation between oral health and longevity, as well as the role of oral health in promoting general health and QOL.

OHRQOL is a comprehensive evaluation of the effects of oral health levels on physical, psychological, and social functions, as well as the effectiveness of the current oral care practices and oral care demands.^[15,16]^ Through the OHIP-14 scale, we found that compared with gingivitis and subgingival calculus, the influence of moderate or severe chronic periodontitis on oral health-related life quality was more obvious, especially up to 11% of severe periodontitis patients who reported an “unsatisfactory diet.” The most common negative effects in gingivitis and subgingival calculus patients were “uncomfortable to eat foods.” Dentition defects also significantly affected the QOL related to oral health, the OHIP-14 score being similar to periodontitis, but the most frequent negative effect was “trouble pronouncing words.” Moreover, in the investigation of all oral diseases, the OHIP-14 score of dentition loss was highest, indicating that the QOL was lowest, and how to improve QOL should be an important research direction of oral medicine. In addition, a logistic regression analysis showed that dental caries and dentition defects significantly correlated with subjects being underweight, a finding which has been reported in previous studies and attributed to inadequate intake of essential amino acids and vitamins owing to avoidance of difficult to chew foods such as fruits, vegetables, and meat.^[17,18]^

In conclusion, our results showed that the oral health of the elderly in Southwest China was relatively poor, compared with the eastern developed areas. Periodontal disease, dental caries, and dentition defects significantly affected the QOL of the elderly. We need to enhance the awareness and behavior of oral healthcare in the elderly, intensify education about oral healthcare, pay more attention to the relationship between general health and oral healthcare, and provide affordable services and professional interventions and treatment measures to improve the oral health status of the elderly in Southwest China in an effective manner.

## Author contributions

**Conceptualization:** Xun Sheng, Xihong Zhang, Hua Zhong.

**Data curation:** Xun Sheng, Xia Xiao, Xiaoxiao Song, Lei Qiao, Xihong Zhang, Hua Zhong.

**Formal analysis:** Xia Xiao, Xiaoxiao Song, Lei Qiao, Xihong Zhang.

**Project administration:** Hua Zhong.

**Validation:** Xun Sheng.

**Writing – original draft:** Xun Sheng, Xia Xiao, Xihong Zhang.

**Writing – review & editing:** Xun Sheng, Hua Zhong.
